# Therapeutic errors involving diabetes medications reported to United States poison centers

**DOI:** 10.1186/s40621-024-00536-y

**Published:** 2024-09-19

**Authors:** Ashley Thurgood Giarman, Hannah L. Hays, Jaahnavi Badeti, Natalie I. Rine, Henry A. Spiller, Motao Zhu, Gary A. Smith

**Affiliations:** 1 Center for Injury Research and Policy, The Abigail Wexner Research Institute at Nationwide Children’s Hospital, 700 Children’s Drive, Columbus, OH 43205 USA; 2 A.T. Still, University of Health Sciences, 5850 E. Still Circle, Mesa, AZ 85206 USA; 3 Central Ohio Poison Center, Nationwide Children’s Hospital, 700 Children’s Drive, Columbus, OH 43205 USA; 4 Department of Pediatrics, The Ohio State University College of Medicine, 1645 Neil Avenue, Columbus, OH 43210 USA; 5 Child Injury Prevention Alliance, PO Box 30545, Columbus, OH USA

**Keywords:** Therapeutic error, Adverse event, National Poison Data System, Diabetes

## Abstract

**Background:**

To investigate the characteristics and trends of therapeutic errors that occur outside of healthcare facilities involving diabetes medications reported to US poison centers.

**Methods:**

National Poison Data System data from 2000 to 2021 were retrospectively analyzed.

**Results:**

There were 157,623 exposure cases of non-healthcare facility-related therapeutic errors associated with diabetes medications as the primary substance reported to US poison centers from 2000 to 2021. The rate of all therapeutic errors involving diabetes medications increased by 279.8% from 2000 to 2011, followed by a slower 15.0% increase to 2021. Half (50.1%) of the exposure cases were treated/evaluated and released and 44.1% did not receive treatment in a healthcare facility; however, 9.9% experienced a serious medical outcome, including 17 fatalities, and 1.0% were admitted to a critical care unit and 2.2% to a non-critical care unit. Insulin had the highest rates of therapeutic errors and serious medical outcomes, while sulfonylureas and insulin had the highest medical hospital admission rates. Metformin accounted for 59% (*n* = 10) of fatalities.

**Conclusions:**

Although most cases of therapeutic errors involving diabetes medications had no or minimal clinical consequences, an important minority were associated with a serious medical outcome or medical hospital admission. Increased efforts to prevent therapeutic errors involving diabetes medications are warranted.

**Supplementary Information:**

The online version contains supplementary material available at 10.1186/s40621-024-00536-y.

## Background

Diabetes is a major public health problem in the United States (US) with 29.7 million people (8.9% of the population) diagnosed with diabetes in 2021 (Center for Disease Control and Prevention (CDC) 2024). Diabetes was listed as a diagnosis for 7.86 million hospital discharges among US adults in 2020. Estimated direct costs of diagnosed diabetes were$307 billion in 2022. Complications from diabetes include heart disease, stroke, neuropathy, retinopathy, hearing loss, and chronic kidney disease. Diabetes was the eighth leading cause of death in the US in 2021, with 103,294 fatalities (Center for Disease Control and Prevention (CDC) 2024). Medications, including insulin, metformin, sulfonylureas, and GLP-1 receptor agonists, are commonly used to help manage diabetes.

Therapeutic errors associated with the use of diabetes medications have the potential to cause serious effects, including hypoglycemia and lactic acidosis, which can result in serious morbidity or fatality (Klein-Schwartz et al. 2016; VonMach et al. 2006; Geller et al. 2014). However, research specifically investigating therapeutic errors in the non-healthcare facility (non-HCF) setting involving diabetes medications is limited. Studies on out-of-HCF therapeutic errors reported to the National Poison Data System (NPDS) have analyzed data from 2000 to 2005 and 2000–2012, including reported cases involving hormones/hormone antagonists; however, these studies did not focus specifically on diabetes medications (Shah et al. 2009; Brophy et al. 2014; Hodges et al. 2018; Magal et al. 2017; Smith et al. 2014). Hodges, et al. found that hormones/hormone antagonists (in particular, insulin and sulfonylureas) were the third most common pharmaceutical category associated with serious medical outcomes attributable to therapeutic errors reported to the NPDS (Hodges et al. 2018). Another study evaluating therapeutic errors among children < 6 years old using NPDS data found that hormones/hormone antagonists were associated with almost 13,000 reported cases from 2000 to 2012 (Smith et al. 2014). Additional studies have been limited to therapeutic errors involving single medication categories, such as only insulin (Spiller et al. 2011; Beuhler et al. 2013) or metformin (Stevens et al. 2019), which precluded comparisons among diabetes medication categories. The objective of this study is to investigate and compare the characteristics and trends of therapeutic errors involving diabetes medications that occur outside of HCFs reported to US poison centers (PCs) from 2000 to 2021.

## Methods

### Data sources

Data for 2000 through 2021 were obtained from the NPDS, which is the data warehouse for calls received by PCs in the US and is managed by America’s Poison Centers (America’s Poison Centers 2024). Population estimates, including age group-specific and sex-specific estimates, were obtained from the US Census Bureau for years 2000 through 2021 and were used to calculate population-based exposure rates (United States Census Bureau  2023). This study was deemed to be exempt from approval by the institutional review board at the authors’ institution.

### Case selection criteria

This study included exposure cases reported to US PCs from January 1, 2000, through December 31, 2021, associated with therapeutic errors involving medications used to manage diabetes that occurred at locations other than a HCF. Cases were identified using NPDS generic codes for these medications. Cases were excluded from the study if they had a medical outcome of “confirmed non-exposure” or “unrelated effect.” Exclusions included three fatalities that had a relative contribution to fatality, as determined by America’s Poison Centers, coded as “probably not responsible” (two individuals) or “clearly not responsible” (one individual).

### Study variables

Study variables included year, age group (< 20, 20–39, 40–49, 50–59, 60–69, 70–79, and > 79 years), sex, medication category, type of exposure (single-substance or multiple-substance exposure), highest level of health care received, medical outcome, and therapeutic error scenario. Medications were categorized as (1) insulin, (2) metformin (i.e., biguanides), (3) sulfonylureas, (4) glucagon-like peptide-1 (GLP-1) receptor agonists, and (5) other (including alpha-glucosidase inhibitors, dipeptidyl peptidase-4 inhibitors, meglitinides, sodium glucose co-transporter 2 inhibitors, thiazolidinediones, biguanide or sulfonylurea medication combinations, and other/unknown single or combination medications). The generic code for GLP-1 receptor agonists was added to the NPDS in December 2015 and new GLP-1 receptor agonist medications were introduced in the NPDS during the study period.

The highest level of health care received, as categorized by the NPDS (America’s Poison Centers 2023), included (1) no HCF treatment received (i.e., managed at home), (2) treated/evaluated and released from a HCF, (3) admitted to a critical care unit (CCU), (4) admitted to a non-CCU, (5) admitted to a psychiatric facility, (6) patient refused referral /did not arrive at a HCF, and (7) unknown (which includes lost to follow-up, left against medical advice, and unknown). If the management site was “unknown,” then that case was included in the unknown category for highest level of health care received. During analyses, “admitted to a CCU” and “admitted to a non-CCU” were combined to represent medical admissions to a HCF.

Medical outcome was categorized according to the NPDS (America’s Poison Centers 2023) as (1) no effect, (2) minor effect (minimally bothersome symptoms that generally resolve rapidly with no residual disability), (3) moderate effect (more pronounced, prolonged, or systemic than minor symptoms), (4) major effect (symptoms are life-threatening or result in significant disability or disfigurement), (5) death, (6) not followed (includes minimal clinical effects possible and judged as a non-toxic exposure), and (7) unable to follow (judged as a potentially toxic exposure). During analyses, moderate effect, major effect, and death were combined into the category, “serious medical outcome.” In addition, during analyses, “unable to follow” was consider as “unknown.”

Deaths reported through the NPDS undergo an extensive formal review process and are given a relative contribution to fatality classification: (1) undoubtedly responsible, (2) probably responsible, (3) contributory, (4) probably not responsible, (5) clearly not responsible, or (6) unknown. America’s Poison Centers considers a fatality to be related to the exposure if its fatality review judges the substance exposure to be at least contributory, which means it has a relative contribution to fatality classification of 1-3 (Gummin 2022).

### Statistical analysis

IBM SPSS 28.0 Statistics (IBM Corporation, Armonk, NY) and SAS 9.4 (SAS Institute, Inc. Cary, NC) were used for data analyses. Except for trend analyses, all analyses were limited to exposure cases involving a diabetes medication as the primary substance (including both single-substance and multiple-substance exposures). A primary substance is the substance that is judged by a specialist in poison information at the reporting PC to be most likely responsible for the observed clinical effects. Trends over time were analyzed using all reported exposure cases (including primary and non-primary substance exposures). Piecewise or simple linear regression models were used to determine the statistical significance of trends by evaluating whether the null hypothesis of slope = 0 could be rejected. Break points for piecewise analyses were determined based on scatter plots. The level of statistical significance was α=0.05. Risk ratios (RRs) with 95% confidence intervals (CIs) were calculated to determine the magnitude of association of several factors with (1) medical admission, (2) serious medical outcome, or (3) death.

## Results

### General characteristics

There were 157,623 exposure cases involving therapeutic errors associated with diabetes medications as the primary substance reported to US PCs from 2000 to 2021, averaging 7,165 therapeutic errors annually (Table 1). The frequency of therapeutic errors increased by 377.1% from 2,083 in 2000 to 9,939 in 2021; there was an average of one therapeutic error reported to a PC each hour in 2021. One-fourth (25.6%) of the therapeutic errors were accounted for by 60-69-year-olds, followed by 50-59-year-olds (21.7%), and 70-79-year-olds (18.1%). Females accounted for more than half (62.2%) of all therapeutic errors, and most (82.1%) therapeutic errors involved a single substance. The proportion of multiple-substance exposures increased with increasing age group (Table 1).

**Table 1:** Characteristics of therapeutic errors Associated with Diabetes medications as the primary substance reported to United States Poison Centers by Age Group, national poison Data System 2000–2021

Characteristics	Age Groups								Total
	< 20 Years	20–39 Years	40–49 Years	50–59 Years	60–69 Years	70–79 years	> 79 Years	Unknown	
	*n* (%)^a^	*n* (%)^a^	*n* (%)^a^	*n* (%)^a^	*n* (%)^a^	*n* (%)^a^	*n* (%)^a^		*n* (%)^a^
**Total (Row %)** ^b^	7,688 (5.3)	13,612 (9.4)	16,503 (11.3)	31,588 (21.7)	37,210 (25.6)	26,299 (18.1)	12,635 (8.7)	12,088	157,623 (100.0)
**Sex**									
Male	4,152 (54.0)	4,882 (35.9)	6,163 (37.4)	11,271 (35.7)	13,803 (37.1)	10,103 (38.4)	4,770 (37.8)	4,377	59,521 (37.8)
Female	3,531 (46.0)	8,723 (64.1)	10,332 (62.6)	20,306 (64.3)	23,399 (62.9)	16,191 (61.6)	7,855 (62.2)	7,624	97,961 (62.2)
Unknown	5	7	8	11	8	5	10	87	141
**Type of exposure**									
Single substance	6,874 (89.4)	11,922 (87.6)	13,888 (84.2)	26,208 (83.0)	30,483 (81.9)	20,625 (78.4)	8,894 (70.4)	10,528	129,422 (82.1)
Multiple substance	814 (10.6)	1,690 (12.4)	2,615 (15.8)	5,380 (17.0)	6,727 (18.1)	5,674 (21.6)	3,741 (29.6)	1,560	28,201 (17.9)
**Highest level of health care received**									
No treatment received in a HCF	4,876 (63.9)	9,659 (76.0)	11,905 (76.5)	22,277 (75.1)	25,782 (73.8)	17,517 (71.2)	7,884 (67.4)	9,015	108,915 (44.1)
Treated/evaluated and released	1,481 (19.4)	1,958 (15.4)	2,360 (15.2)	4,830 (16.3)	6,057 (17.3)	4,429 (18.0)	2,009 (17.2)	723	123,847 (50.1)
Admitted to a healthcare facility	751 (9.8)	550 (4.3)	620 (4.0)	1,266 (4.3)	1,677 (4.8)	1,611 (6.6)	1,245 (10.7)	112	7,869 (3.2)
Critical care unit	214 (2.8)	192 (1.5)	185 (1.2)	375 (1.3)	480 (1.4)	477 (1.9)	337 (2.9)	37	2,337 (1.0)
Non-critical care unit	537 (7.0)	358 (2.8)	435 (2.8)	891 (3.0)	1,197 (3.4)	1,134 (4.6)	908 (7.8)	72	5,532 (2.2)
Psychiatric facility	4 (0.1)	10 (0.1)	12 (0.1)	13 (0.0)	8 (0.0)	3 (0.0)	2 (0.0)	3	55 (0.0)
Patient refused referral/ Did not arrive at HCF	195 (2.5)	527 (4.1)	667 (4.3)	1,288 (4.3)	1,421 (4.1)	1,035 (4.2)	549 (4.7)	609	6,291 (2.5)
Unknown ^c^	381	881	939	1,914	2,265	1,704	906	1,629	10,646
**Medical outcome**									
No effect	2,683 (37.2)	3,697 (29.7)	5,301 (34.9)	11,591 (39.9)	14,947 (43.6)	11,114 (46.1)	5,207 (45.3)	2,805	57,345 (39.9)
Minor effect	520 (7.2)	998 (8.0)	1,158 (7.6)	1,976 (6.8)	2,089 (6.1)	1,361 (5.6)	609 (5.3)	403	9,114 (6.3)
Serious medical outcome^d^	710 (9.8)	1,194 (9.6)	1,383 (9.1)	2,742 (9.4)	3,329 (9.7)	2,745 (11.4)	1,602 (13.9)	520	14,225 (9.9)
Moderate effect	677 (9.4)	1,154 (9.3)	1,340 (8.8)	2,651 (9.1)	3,215 (9.4)	2,643 (10.9)	1,535 (13.3)	507	13,722 (9.6)
Major effect	33 (0.5)	39 (0.3)	41 (0.3)	91 (0.3)	109 (0.3)	94 (0.5)	66 (0.6)	13	486 (0.3)
Death	0 (0.0)	1 (0.0)	2 (0.0)	0 (0.0)	5 (0.0)	8 (0.0)	1 (0.0)	0	17 (0.0)
Not followed^e^	3,301 (45.8)	6,550 (52.7)	7,366 (48.4)	12,716 (43.8)	13,907 (40.6)	8,911 (36.9)	4,085 (35.5)	6,161	62,997 (43.8)
Unable to follow^f^	462	1,173	1,295	2,563	2,938	2,168	1,132	2,199	13,942
**Medication category**									
Insulin	1,998 (26.0)	4,979 (36.6)	6,770 (41.0)	15,119 (47.9)	19,369 (52.1)	13,722 (52.2)	5,998 (47.5)	5,888	73,843 (46.8)
Metformin	3,652 (47.5)	5,870 (43.1)	5,708 (34.6)	9,043 (28.6)	9,419 (25.3)	5,744 (21.8)	2,386 (18.9)	3,653	45,475 (28.8)
Sulfonylureas	1,214 (15.8)	1,487 (10.9)	2,020 (12.2)	3,836 (12.1)	4,621 (12.4)	4,327 (16.5)	3,045 (24.1)	1,099	21,649 (13.7)
GLP-1 receptor agonists	25 (0.3)	278 (2.0)	462 (2.8)	859 (2.7)	800 (2.2)	433 (1.6)	93 (0.7)	314	3,264 (2.1)
Other	799 (10.4)	998 (7.3)	1,543 (9.3)	2,731 (8.6)	3,001 (8.1)	2,073 (7.9)	1,113 (8.8)	1,134	13,392 (8.5)

*GLP-1* glucagon-like peptide-1, *HCF* healthcare facility

^a^ Column percentage may not add to 100.0% due to rounding error

^b^ Row percentages do not add to 100.0% due to rounding error

^c^ Unknown = patient lost to follow-up, left against medical advice, or unknown

^d^ Serious medical outcome is a category that combines the categories of moderate effect, major effect, and death

^e^ Not followed (includes minimal clinical effects possible and judged as a non-toxic exposure)

^f^ Unable to follow (judged as a potentially toxic exposure)

### Medical outcome and highest level of health care received

Most therapeutic errors were “not followed (minimal clinical effects possible or judged as non-toxic exposure)” (43.8%) or experienced “no effect” (39.9%) (Table 1). Half of the therapeutic errors were “treated/evaluated and released” (50.1%) and 44.1% did not receive treatment in a HCF (44.1%). However, 9.9% of therapeutic errors experienced a serious medical outcome. Therapeutic errors were associated with admission to a CCU (1.0%) or non-CCU (2.2%) in 3.2% of cases.

Individuals 50 years or older accounted for almost three-fourths of the serious medical outcomes (73.2%) and admissions to a CCU or non-CCU (73.4%) associated with therapeutic errors (Table 1). Compared with younger individuals, this age group was more likely to experience a serious medical outcome (RR: 1.11; 95% CI: 1.07–1.16) or medical admission (RR: 1.07; 95% CI: 1.02–1.13). Most serious medical outcomes (80.4%) and medical admissions (70.0%) were associated with single-substance exposures. However, multiple-substance exposures were more likely to result in a serious medical outcome (RR: 1.11; 95% CI: 1.07–1.15) or medical admission (RR: 1.95; 95% CI: 1.86–2.04) than single-substance exposures.

There were 17 fatalities associated with therapeutic errors during the study period, including 10 (59%) among females. Ten deaths (59%) were associated with metformin, followed by sulfonylureas (24%, *n* = 4), insulin (12%, *n* = 2), and “other/unknown” (6%, *n* = 1). America’s Poison Centers determined that the relative contribution to fatality of the diabetes medication was “undoubtedly responsible” in two (12%) cases, “probably responsible” in three (18%), “contributory” in three (18%), and unknown in nine (53%) cases. Almost half (47%, *n* = 8) of fatalities occurred among 70-79-year-olds, followed by 29% (*n* = 5) among 60-69-year-olds. The exposure was “chronic” in 10 (59%) cases, “acute-on-chronic” in four (24%), “acute” in one (6%), and “unknown chronicity” in two (12%) cases. Hypoglycemia was reported for all sulfonylurea and insulin-related fatalities, and for two (20%) of the metformin-related deaths. Acidosis was reported in all 10 metformin-related fatalities, with the acidosis judged to be related to metformin in eight (80%) of those cases and unknown if related in two (20%) cases. Although 53% (*n* = 9) of the deaths were among single-substance exposures, multiple-substance exposures were more likely to be associated with fatality (RR: 4.04, 95% CI: 1.56–10.46) than single-substance exposures. Compared with insulin, metformin (RR: 7.27, 95% CI: 1.59–33.16) and sulfonylureas (RR: 6.81, 95% CI: 1.25–37.17) were more likely to be associated with death; however, the association with fatality was similar for metformin compared with sulfonylureas (RR: 1.07, 95% CI: 0.33–3.40).

### Medication category

Most therapeutic errors were associated with insulin (46.8%), followed by metformin (28.8%), and sulfonylureas (13.7%) (Table 1). Although the proportion of insulin-related therapeutic errors generally increased with increasing age group, there was a decrease among individuals 80 years or older. In contrast, the proportion of metformin-related therapeutic errors decreased with increasing age group. Although metformin accounted for more therapeutic errors than insulin among individuals in the < 20 years old and 20–39 years old age groups, insulin was the most common medication associated with therapeutic errors among age groups greater than 39 years old.

Approximately two-thirds (65.7%) of the serious medical outcomes were associated with therapeutic errors involving insulin, followed by sulfonylureas (23.5%) and metformin (6.6%) (Table 1). However, among the 73,843 insulin exposures, almost half (46.4%) were associated with no effect (Table 2). Similarly, 36.9% of the 21,649 sulfonylurea exposures were associated with no effect. Compared with metformin, sulfonylureas (RR: 8.28, 95% CI: 7.72–8.89) and insulin (RR: 6.81, 95% CI: 6.37–7.27) were more likely to be associated with a serious medical outcome. In addition, sulfonylureas were more likely than insulin (RR: 1.22, 95% CI: 1.17–1.26) to be associated with a serious medical outcome. Likewise, compared with metformin, sulfonylureas (RR: 10.68, 95% CI: 9.86–11.57) and insulin (RR: 3.25, 95% CI: 2.99–3.52) were more likely to be associated with medical admission. In addition, sulfonylureas were more likely than insulin (RR: 3.29, 95% CI: 3.14–3.44) to be associated with medical admission.

**Table 2:** Medical outcome of therapeutic errors Associated with Diabetes medications as the primary substance reported to United States Poison Centers by Medication Category, national poison Data System 2000–2021

Medication categories	Medical outcome								Total *n* (%)^d^
	No effect *n* (%)^d^	Minor effect *n* (%)^d^	Moderate effect *n* (%)^d^	Major effect *n* (%)^d^	Death *n* (%)^d^	Serious medical outcome^a^ *n* (%)^d^	Not followed^b^ *n* (%)^d^	Unable to follow^c^ *n*	
Insulin	34,228 (59.7)	4,077 (44.7)	9,122 (66.5)	215 (44.2)	2 (11.9)	9,339 (65.7)	16,776 (26.6)	9,423	73,843 (46.8)
Metformin	9,938 (17.3)	2,579 (28.3)	885 (6.4)	49 (10.1)	10 (58.8)	944 (6.6)	30,867 (49.0)	1,147	45,475 (28.9)
Sulfonylureas	7,985 (13.9)	1,437 (15.8)	3,140 (22.9)	194 (39.9)	4 (23.5)	3,338 (23.5)	6,162 (9.8)	2,727	21,649 (13.7)
GLP-1 receptor agonists	774 (1.3)	402 (4.4)	143 (1.0)	7 (1.4)	0 (0.0)	150 (1.1)	1,779 (2.8)	159	3,264 (2.1)
Other	4,420 (7.7)	619 (6.8)	432 (3.1)	21 (4.3)	1 (5.9)	454 (3.2)	7,413 (11.8)	486	13,392 (8.5)
Total (Row%)^e^	57,345 (39.9)	9,114 (6.3)	13,722 (9.6)	486 (0.3)	17 (0.0)	14,225 (9.9)	62,997 (43.8)	13,942	157,623 (100.0)

*GLP-1* glucagon-like peptide-1

^a^Serious medical outcome includes moderate effect, major effect, and death combined

^b^ Not followed (includes minimal clinical effects possible and judged as a non-toxic exposure)

^c^ Unable to follow (judged as a potentially toxic exposure)

^d^ Column percentage may not add to 100.0% due to rounding error

^e^ Row percentages do not add to 100.0% due to rounding error

.

### Type of therapeutic error

The most common therapeutic error scenario was “wrong medication taken/given” (30.6%), followed by “inadvertently took/given medication twice” (29.1%), “other incorrect dose” (13.3%), and “inadvertently took/given someone else’s medication” (9.8%) (Table 3). The relative frequency of these scenarios showed some variation by age group. For example, among individuals < 20 years old, “inadvertently took/given someone else’s medication” (39.2%) was the most common scenario.

**Table 3:** Top ten therapeutic error scenarios involving diabetes medications as the primary substance reported to United States Poison Centers, national poison Data System 2000–2021

Scenarios	Total
	*n* (%)^a^
Wrong medication taken/given	48,263 (30.6)
Inadvertently took/given medication twice	45,824 (29.1)
Other incorrect dose	20,970 (13.3)
Inadvertently took/given someone else’s medication	15,432 (9.8)
Medication doses given/taken too close together	9,580 (6.1)
Incorrect formulation or concentration given	8,187 (5.2)
Other/unknown therapeutic error	7,986 (5.1)
Confused units of measure	3,259 (2.1)
Healthcare professional iatrogenic error	1,736 (1.1)
Incorrect formulation or concentration dispensed	1,318 (0.8)

^a^Column percentages were calculated by dividing the number of times a scenario was associated with a case by the number of cases with at least one scenario (*n* = 157,563; 60 cases had no scenario listed). Percentages will not sum to 100.0% because some cases had more than one reported scenario and not all scenarios are included in the table

### Trends

**Fig. 1:**
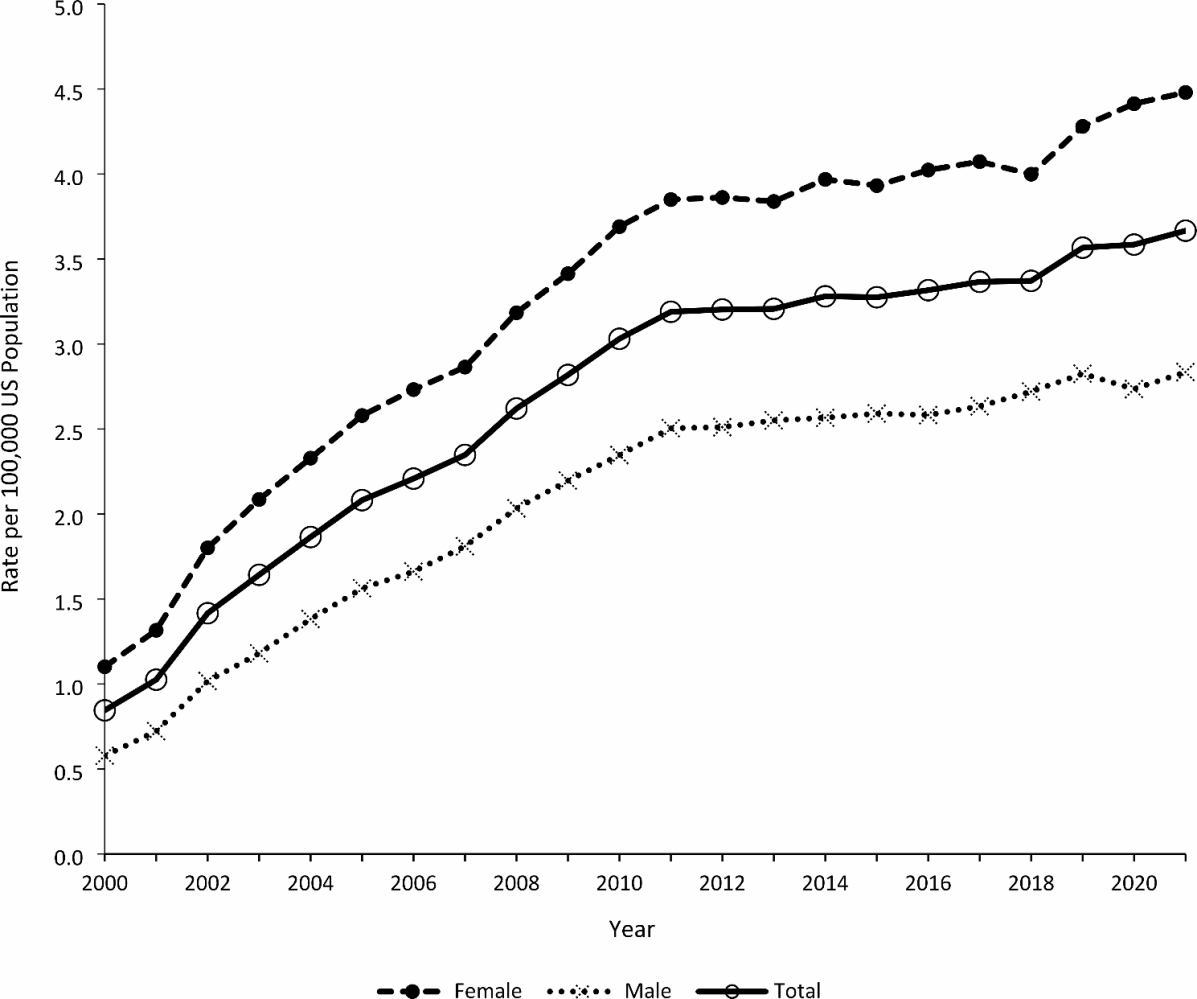
Annual Rate of Therapeutic Errors Involving Diabetes Medications Reported to United States Poison Centers by Sex, National Poison Data System 2000–2021

Trend analyses included all 184,897 therapeutic error exposure cases involving diabetes medications, regardless of whether they were judged to be the primary substance. The annual rate of therapeutic errors involving diabetes medications per 100,000 US population increased by 279.8% from 0.84 in 2000 to 3.19 in 2011 (*P* < 0.0001), followed by a slower 15.0% increase to 3.67 in 2021 (*P* < 0.0001) (Fig. 1). Females and males demonstrated similar increasing trends, with females experiencing higher rates of therapeutic errors than males. The difference between female and male rates widened during the study period.

**Fig. 2:**
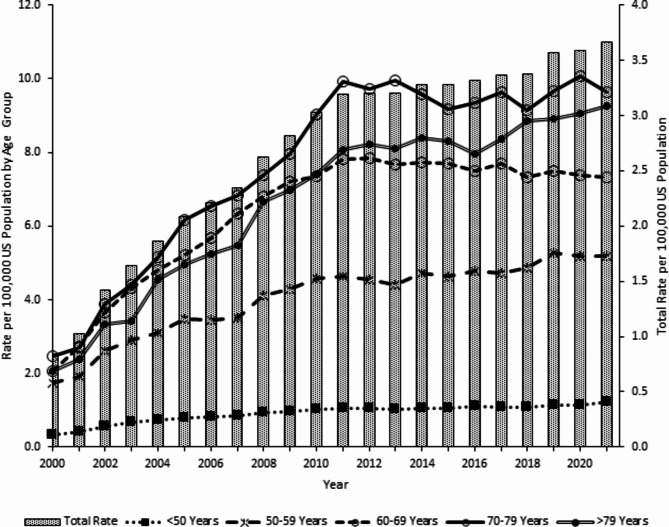
Annual Rate of Therapeutic Errors Involving Diabetes Medications Reported to United States Poison Centers by Age Group, National Poison Data System 2000–2021

Although all age groups demonstrated therapeutic error rate increases during the study period, older adults 60 years or older drove the overall trend pattern, with 70-79-year-olds experiencing the highest rates (Fig. 2). The therapeutic error rate among 60-69-year-olds increased by 277.8% from 2.07 in 2000 to 7.82 in 2011 (*P* < 0.0001), followed by a 6.3% decrease to 7.33 in 2021 (*P* = 0.0003). The rate of therapeutic errors among 70-79-year-olds increased by 302.0% from 2.47 in 2000 to 9.93 in 2011 (*P* < 0.0001), and then plateaued with a rate of 9.63 in 2021 (*P* = 0.8638). Among individuals > 79 years old, the therapeutic error rate increased by 347.8% from 2.07 in 2000 to 9.27 in 2021 (*P* < 0.0001).

**Fig. 3:**
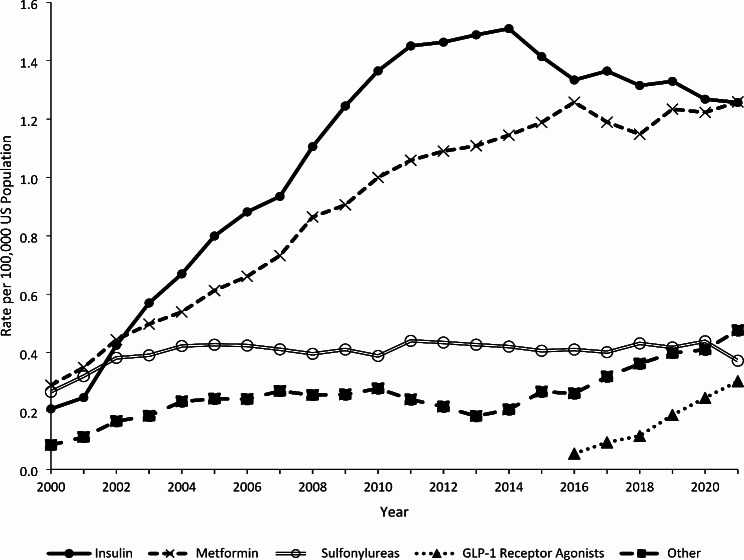
Annual Rate of Therapeutic Errors Involving Diabetes Medications Reported to United States Poison Centers by Medication Category, National Poison Data System 2000–2021

Insulin and metformin demonstrated the highest therapeutic error rates, starting in 2002 (Fig. 3). The rate of insulin-related therapeutic errors increased by 619.0% from 0.21 in 2000 to 1.51 in 2014 (*P* < 0.0001), followed by a 16.6% decrease to 1.26 in 2021 (*P* < 0.0001). The rate associated with metformin increased by 334.5% from 0.29 in 2000 to 1.26 in 2021 (*P* < 0.0001). The rate for sulfonylureas increased 55.6% from 0.27 in 2000 to 0.42 in 2004 (*P* < 0.0001) before plateauing to 0.37 in 2021 (*P* = 0.3757). The rate of therapeutic errors associated with GLP-1 receptor agonists increased by 500.0% from 0.05 in 2016 to 0.30 in 2021 (*P* = 0.0002).

The rate of serious medical outcomes associated with therapeutic errors involving diabetes medications increased 383.3% from 0.06 in 2000 to 0.29 in 2010 (*P* < 0.0001) and then plateaued to 0.30 in 2021 (*P* = 0.9172) (Appendix 1). The rate of therapeutic error-related medical admissions increased by 425.0% from 0.04 in 2000 to 0.21 in 2021 (*P* < 0.0001).

Compared with all other diabetes medications, insulin had the highest rate of serious medical outcomes associated with therapeutic errors starting in 2002 (Appendix 2). Its rate increased by 850.0% from 0.02 in 2000 to 0.19 in 2010 (*P* < 0.0001), followed by a 21.1% decrease to 0.15 in 2021 (*P* < 0.0001). The rate for sulfonylureas increased 100.0% from 0.03 in 2000 to 0.06 in 2005 (*P* < 0.0001) and then plateaued to 0.06 in 2021 (*P* = 0.0629). The rate for metformin increased by 400.0% from 0.01 in 2000 to 0.05 in 2021 (*P* < 0.0001). The rate of serious medical outcomes associated with GLP-1 receptor agonist-related therapeutic errors increased by 300.0% from 0.004 in 2016 to 0.016 in 2021 (*P* = 0.0111).

Therapeutic errors associated with insulin and sulfonylureas had the highest rates of medical admission during the study period (Appendix 3). The rate for insulin increased by 1,216.7% from 0.006 in 2000 to 0.079 in 2021 (*P* < 0.0001), and the rate for sulfonylureas increased by 187.0% from 0.023 in 2000 to 0.066 in 2021 (*P* < 0.0001). The rate for metformin also increased by 400.0% from 0.007 in 2000 to 0.035 in 2021 (*P* < 0.0001). The rate of medical admission associated with GLP-1 receptor agonist-related therapeutic errors increased 500.0% from 0.002 in 2016 to 0.012 in 2021 (*P* = 0.0018).

## Discussion

The rate of therapeutic errors that occurred outside of HCFs involving diabetes medications reported to US PCs increased by approximately 280% from 2000 to 2011, followed by a slower 15% increase to 2021. Consistent with previous research (Magal et al. 2017; Spiller et al. 2011), two-thirds of the therapeutic errors in this study were among individuals 60 years or older. Rates among these older age groups increased more than those among younger individuals during the study period, driving the observed overall rate trend for therapeutic errors. The female predominance (> 60%) in this study also was consistent with prior studies (Magal et al. 2017; Spiller et al. 2011). Although most therapeutic errors did not result in clinically important consequences, 9.9% were associated with a serious medical outcome and 3.2% with medical admission to a HCF. The trend pattern for insulin-related therapeutic errors was similar to that among individuals 60 years and older. These trends may have been influenced by the rate of diagnosed diabetes in the US, which increased among adults from 2000 to 2009 before decreasing through 2021 (Centers for Disease Control and Prevention (CDC) 2024). Variation of therapeutic errors by age group related to the different categories of diabetes medications may have been influenced by the different treatment regimens used for diabetes among individuals of different ages. Although investigating the rate trends of prescriptions for diabetes medications was beyond the scope of this study, metformin prescribing trends in the US between 2000 and 2015 have been evaluated using the Medical Expenditure Panel Survey (Le et al. 2019). The metformin prescription rate pattern showed a decrease for 2006–2008, followed by a large increase from 2008 to 2011, which does not match the steady increase in the rate of metformin-related therapeutic errors during that time period in our study.

Compared with other diabetes medications, insulin was associated with the highest rate of therapeutic errors reported to PCs in this study (starting in 2002), although previous research indicates that most of these incidents can be managed at home (Beuhler et al. 2013). Insulin also had the highest rate of serious medical outcomes, which corroborates previous research (VonMach et al. 2006). While the annual rate of insulin-related serious medical outcomes increased rapidly until 2010 before plateauing and then decreasing to 2021, the rate of insulin-related medical admissions to a hospital did not reverse in 2010 and continued to rise throughout the study period. Both insulin and sulfonylureas are among the medications most commonly associated with hospital admission among older adults (Budnitz et al. 2011). Because insulin is used in small doses and has a narrow therapeutic window, there is little margin for error (Klein-Schwartz et al. 2016). The most common serious adverse effect of insulin is hypoglycemia. Unger found that the prevalence of hypoglycemic episodes was higher for insulin (7.3%) than sulfonylureas (0.8%) (Unger 2012).

Although the rate of sulfonylurea-related serious medical outcomes in this study was lower than that of insulin, sulfonylureas and insulin had comparable rates of medical admission to a hospital starting in 2008. Medical admission was more likely for individuals with therapeutic errors associated with sulfonylureas than those associated with insulin or with metformin. These findings may be explained by the well-known delayed onset of action of sulfonylureas, especially among children, which appropriately often results in precautionary hospital admission to monitor for delayed hypoglycemia.

Although metformin was associated with only 29% of the therapeutic error cases in this study, it accounted for 59% (*n*= 10) of fatalities. This is consistent with findings from von Mach and colleagues (VonMach et al. 2006). Hypoglycemia and lactic acidosis have been reported in about 10% and one-third of metformin overdoses, respectively (Klein-Schwartz et al. 2016). Fatality rates associated with metformin-associated lactic acidosis and metformin-induced lactic acidosis can be high and are often characterized by central nervous system effects, kidney failure, and cardiovascular collapse (Spiller et al. 2006). Our findings corroborate those in the literature, with 20% (*n*= 2) of metformin-related deaths with documented hypoglycemia and 100% with documented acidosis. Metformin clearance varies among individuals and those with poor metformin clearance may not tolerate the dose increase associated with therapeutic errors, which then may result in lactic acidosis (Dyatlova 2024). Most of the fatalities in our study were associated with chronic (*n* = 7) or acute-on-chronic (*n* = 2) exposure.

GLP-1 receptor agonists are a medication class approved for management of diabetes and obesity. As their use has rapidly increased in recent years, so have reports to US PCs involving these medications. One study found that from 2021 to 2022, the rate of reported cases to PCs increased by 81%, and of the exposures involving GLP-1 receptor agonists reported from 2017 to 2022, 80% were related to therapeutic errors (Gaw et al. 2024). In our study, GLP-1 receptor agonists accounted for only 2% of the therapeutic error cases but the rate of reported therapeutic errors associated with these medications increased rapidly from 2016 to 2021. This increase is likely related to an increase in prescribing and use of these agents as well as the new GLP-1 receptor agonists that were introduced in the NPDS during the study period. These therapeutic error incidents tended to be less severe with only 4.8% of these cases associated with serious medical outcomes compared with 9.9% for all diabetes medications combined.

The most common therapeutic error scenarios in this study were “wrong medication taken/given,” “inadvertently took/given medication twice,” “other incorrect dose,” and “inadvertently took/given someone else’s medication.” This is consistent with the findings from Geller, et al. that found that “administration of the wrong insulin product” was the most common therapeutic error associated with emergency department visits involving insulin-related hypoglycemia and therapeutic errors (Geller et al. 2014). It is also consistent with previous research that investigated therapeutic errors associated with all pharmaceutical substances, which found that “inadvertently took/given medication twice” was the most common scenario (Brophy et al. 2014). However, our study revealed that therapeutic errors among individuals < 20 years old were most commonly attributed to “inadvertently took/given someone else’s medication,” which differs with findings from Smith, et al., who reported that “inadvertently took/given medication twice” was the most common scenario among children < 6 years old for all pharmaceutical substances combined (Smith et al. 2014). This may be attributable to the higher prevalence of diabetes among older age groups, which means that therapeutic errors involving diabetes medications among the pediatric population are more likely to be medications not prescribed for them.

Strategies to decrease the leading types of therapeutic errors observed in this study include pill organizers and patient and caregiver education about the use of tracking systems or written/electronic records regarding when medications are taken/given (Hodges et al. 2018). Pill organizers should be child-resistant to help prevent unintended access by children and should be easy to use by older adults. Another strategy is the use of unit-dose packaging, like blister packs, rather than pill bottles. Unit-dose packaging has been shown to improve medication adherence and may help patients and caregivers remember whether a medication has been taken/given (Conn et al. 2015). However, blister packs can be difficult for some older adults to open, which may decrease medication compliance in that population. Injectable medications, such as insulin and GLP-1 receptor agonists, pose additional challenges for prevention of therapeutic errors. Furthermore, strategies to improve medication adherence have been shown to be less effective among older adults and individuals with decreased cognitive function (Conn et al. 2015).

### Study limitations

This study underestimates the frequency of therapeutic errors involving diabetes medications because not all these incidents are reported to PCs. In addition, reported cases may not be representative of the spectrum of therapeutic errors involving diabetes medications in the general population because there may be reporting bias; for example, more serious incidents or those involving young children may be more likely to be reported. Because data are self-reported, PCs and America’s Poison Centers cannot completely verify the accuracy of the information. Exposures reported to PCs do not necessarily represent a poisoning or overdose. Although data management protocols and quality checks are used by PCs, miscoding and mis-categorization may still occur. Mis-categorization may occur in multiple-substance exposures when determining which substance should be considered the primary substance. The NPDS does not indicate whether a diabetes medication was being used for Type 1 or Type 2 diabetes, or some other indication, such as weight loss for a GLP-1 receptor agonist. The insulin medication category included both short-acting and long-acting insulins, and the observation period for each may differ and influence the associated level of care received. Despite these limitations, the NPDS is a comprehensive national database commonly used for epidemiologic investigations of the adverse effects of medications.

## Conclusions

The rate of therapeutic errors that occur outside of HCFs involving diabetes medications reported to US PCs increased by 280% from 2000 to 2011, followed by a slower 15% increase to 2021, and two-thirds of therapeutic errors were among individuals 60 years or older. Although most of these incidents had no or minimal clinical consequences, an important minority of cases were associated with a serious medical outcome or medical admission to a hospital. Starting in 2002, insulin had the highest rate of therapeutic errors and the highest rate of serious medical outcomes in this study. Sulfonylureas and insulin had the highest rates of medical admission to a hospital, which were comparable starting in 2008. Although metformin was associated with only 29% of the therapeutic error cases in this study, it accounted for most (59%) fatalities. Although therapeutic errors associated with GLP-1 receptor agonists were less severe, the reported rate of these incidents has been increasing rapidly in recent years. These findings indicate that increased efforts to prevent therapeutic errors involving diabetes medications, especially among older adults, are warranted.

## Electronic supplementary material

Supplementary Material 1

## Data Availability

No datasets were generated or analysed during the current study.

## Abbreviations

CCU: Critical Care Unit
CI: Confidence Interval
GLP-1: Glucagon–Like Peptide–1
HCF: Healthcare Facility
NPDS: National Poison Data System
PC: Poison Center
RR: Risk Ratio
US: United States
