# Supplementary material for: Therapeutic errors involving diabetes medications reported to United States poison centers
Source: Inj Epidemiol. 2024 Sep 19;11:51. doi: 10.1186/s40621-024-00536-y (PMC11412010; doi:10.1186/s40621-024-00536-y)
Supplement: Supplementary file 1 — Supplementary Material 1 [file 40621_2024_536_MOESM1_ESM.docx]

**Appendix 1. Annual Rate of Therapeutic Errors Involving Diabetes Medications Reported to United States Poison Centers Associated with a Serious Medical Outcome or Admission to a Critical Care Unit or Non-Critical Care Unit, National Poison Data System 2000-2021**

**Appendix 2. Annual Rate of Therapeutic Errors Involving Diabetes Medications Reported to United States Poison Centers Associated with a Serious Medical Outcome by Medication Category, National Poison Data System 2000-2021**

**Appendix 3. Annual Rate of Therapeutic Errors Involving Diabetes Medications Reported to United States Poison Centers Associated with Admission to a Critical Care Unit or Non-Critical Care Unit by Medication Category, National Poison Data System 2000-2021**
